# Host Lipid Transport Protein ORP1 Is Necessary for Coxiella burnetii Growth and Vacuole Expansion in Macrophages

**DOI:** 10.1128/msphere.00104-23

**Published:** 2023-04-05

**Authors:** Baleigh Schuler, Margaret Sladek, Stacey D. Gilk

**Affiliations:** a Department of Microbiology and Immunology, Indiana University School of Medicine, Indianapolis, Indiana, USA; b Department of Pathology and Microbiology, University of Nebraska Medical Center, Omaha, Nebraska, USA; University of Kentucky College of Medicine

**Keywords:** *Coxiella*, ORP, membrane contact sites, cholesterol, intracellular pathogen

## Abstract

Coxiella burnetii is an intracellular bacterium that causes the human disease Q fever. C. burnetii forms a large, acidic *Coxiella*-containing vacuole (CCV) and uses a type 4B secretion system to secrete effector proteins into the host cell cytoplasm. While the CCV membrane is rich in sterols, cholesterol accumulation in the CCV is bacteriolytic, suggesting that C. burnetii regulation of lipid transport and metabolism is critical for successful infection. The mammalian lipid transport protein ORP1L (oxysterol binding protein-like protein 1 Long) localizes to the CCV membrane and mediates CCV-endoplasmic reticulum (ER) membrane contact sites. ORP1L functions in lipid sensing and transport, including cholesterol efflux from late endosomes and lysosomes (LELs), and the ER. Its sister isoform, ORP1S (oxysterol binding protein-like protein 1 Short) also binds cholesterol but has cytoplasmic and nuclear localization. In ORP1-null cells, we found that CCVs were smaller than in wild-type cells, highlighting the importance of ORP1 in CCV development. This effect was consistent between HeLa cells and murine alveolar macrophages (MH-S cells). CCVs in ORP1-null cells had higher cholesterol content than CCVs in wild-type cells at 4 days of infection, suggesting ORP1 functions in cholesterol efflux from the CCV. While the absence of ORP1 led to a C. burnetii growth defect in MH-S cells, there was no growth defect in HeLa cells. Together, our data demonstrated that C. burnetii uses the host sterol transport protein ORP1 to promote CCV development, potentially by using ORP1 to facilitate cholesterol efflux from the CCV to diminish the bacteriolytic effects of cholesterol.

**IMPORTANCE** Coxiella burnetii is an emerging zoonotic pathogen and bioterrorism threat. No licensed vaccine exists in the United States, and the chronic form of the disease is difficult to treat and potentially lethal. Postinfectious sequelae of C. burnetii infection, including debilitating fatigue, place a significant burden on individuals and communities recovering from an outbreak. C. burnetii must manipulate host cell processes in order to promote infection. Our results establish a link between host cell lipid transport processes and C. burnetii’s avoidance of cholesterol toxicity during infection of alveolar macrophages. Elucidating the mechanisms behind bacterial manipulation of the host will yield insight for new strategies to combat this intracellular pathogen.

## INTRODUCTION

Coxiella burnetii, a Gram-negative, rod-shaped bacterium, is the causative agent of the human disease Q fever. C. burnetii is typically transmitted to humans through contact with contaminated aerosols from infected livestock animals (1). While over half of acute Q fever infections are asymptomatic, symptomatic cases cause a febrile illness lasting 10 to 14 days and can require hospitalization in more severe cases (1, 2). The majority of acute Q fever patients recover and clear the infection, but a substantial portion of survivors will develop long-term sequelae, such as Q fever fatigue syndrome (3–5). Recent studies from the 2007–2010 C. burnetii outbreak in the Netherlands have shown persistently decreased quality of life and increased disability among Q fever patients years after recovery (6). Chronic Q fever affects <5% of those infected and manifests months to years after the initial infection as a severe, difficult-to-treat endocarditis or vasculitis, typically in patients with underlying valvulopathies (2, 7). Chronic Q fever treatment involves a minimum of 18 months of antibiotic therapy, with around 20% of chronic Q fever patients succumbing to the disease despite this intensive regimen (1, 2). Without treatment, chronic Q fever is usually fatal (2, 7). Q fever infection is challenging to prevent and treat, with no licensed vaccine in the United States.

As an obligate intracellular pathogen, C. burnetii must establish a replicative niche within the host cell. After phagocytosis, C. burnetii hijacks the host endolysosomal system in order to form the *Coxiella*-containing vacuole (CCV), a large, acidic vacuole that readily fuses with host endosomes, lysosomes, and autophagosomes (8, 9). Within this niche, C. burnetii employs a type 4B secretion system (T4BSS) to secrete effector proteins into the host cell cytoplasm, where they modulate host cell processes to promote bacterial survival and growth (10). The CCV membrane is rich in sterols (11), and while it was initially thought that CCV cholesterol was necessary for C. burnetii growth (11), a cholesterol-free cell model system demonstrated C. burnetii growth actually increases in the absence of cholesterol (12). Intriguingly, cholesterol addition to this system inhibited C. burnetii growth in a dose-dependent manner by increasing CCV acidity and proteolytic activity (12). Despite its sensitivity to cholesterol, C. burnetii growth in cholesterol-rich macrophages suggests that the bacteria employ mechanisms to mitigate cholesterol toxicity. One such strategy may be subversion of host lipid transport proteins at the CCV membrane. We recently demonstrated that, through the T4BSS, C. burnetii actively recruits the eukaryotic sterol binding protein ORP1L (oxysterol binding protein-related protein 1 long) to the CCV membrane (13).

ORP1 is a highly conserved lipid transport protein that has two transcript variants: full-length ORP1L and truncated ORP1S (oxysterol binding protein-related protein 1 short) (14). In eukaryotic cells, ORP1L mediates cholesterol transport from late endosomes and lysosomes (LELs) to the endoplasmic reticulum (ER) at membrane contact sites (MCS) formed through interaction between ORP1L and the ER proteins VAPA and VAPB (15–17). ORP1L and its sister isoform ORP1S, together referred to here as ORP1, share a lipid binding domain (ORD) which binds and transports cholesterol, oxysterols, and phospholipids (14). However, only full-length ORP1L contains the ankyrin repeat domains necessary for LEL and CCV localization and the FFAT motif necessary for LEL-ER membrane contact sites (15, 18). ORP1S localizes to the cytoplasm and nucleus and is not known to participate in membrane contact sites. Based on ORP1L localization at CCV-ER membrane contact sites and the known cholesterol transport ability of ORP1, we hypothesized that C. burnetii uses ORP1 to promote bacterial pathogenesis through cholesterol efflux from the CCV.

In this study, we observed significantly attenuated CCV expansion in ORP1-null HeLa cells, suggesting ORP1 is necessary for normal CCV development. CCVs in ORP1-null cells had higher sterol content than CCVs in wild-type cells, supporting our hypothesis that ORP1 promotes cholesterol efflux from the CCV. C. burnetii growth in HeLa cells was not affected by the absence of ORP1. However, both CCV expansion and C. burnetii growth were inhibited in ORP1-deficient murine alveolar macrophage (MH-S) cells. Taken together, these results demonstrated that during C. burnetii infection, ORP1 promotes CCV development. Our findings support the hypothesis that ORP1 plays a role in cholesterol efflux from the CCV, but discrepancies between ORP1’s effect on C. burnetii growth in differing cell models suggest that in some cell types, compensatory cholesterol transport mechanisms may be sufficient to sustain C. burnetii growth in the absence of ORP1.

## RESULTS

### ORP1 is critical for expansion of the *Coxiella*-containing vacuole.

**FIG 1:**
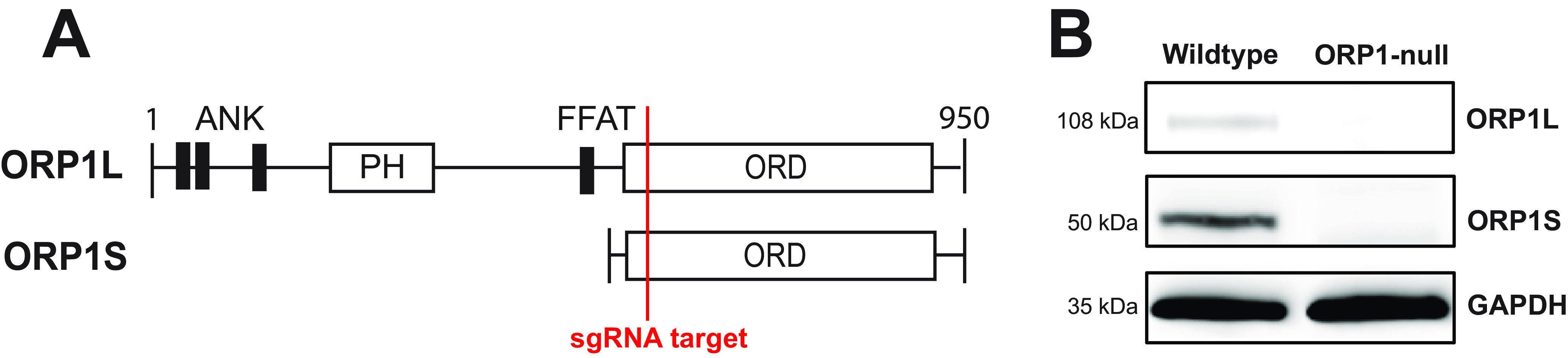
Generation of ORP1-null HeLa cell line. (A) Schematic representation of ORP1 isoforms ORP1L and ORP1S, showing location targeted by sgRNA used in CRISPR-Cas9 generation of ORP1-null cells. ANK, ankyrin repeat domains; FFAT, two phenylalanines in an acidic tract motif; ORD, ORP-family cholesterol binding domain. (B) ORP1 knockout was confirmed by Western blotting. Absence of ORP1L (108 kDa) and ORP1S (50 kDa) was demonstrated in ORP1-null HeLa cells. GAPDH (35 kDa) was used as a loading control.

**FIG 2:**
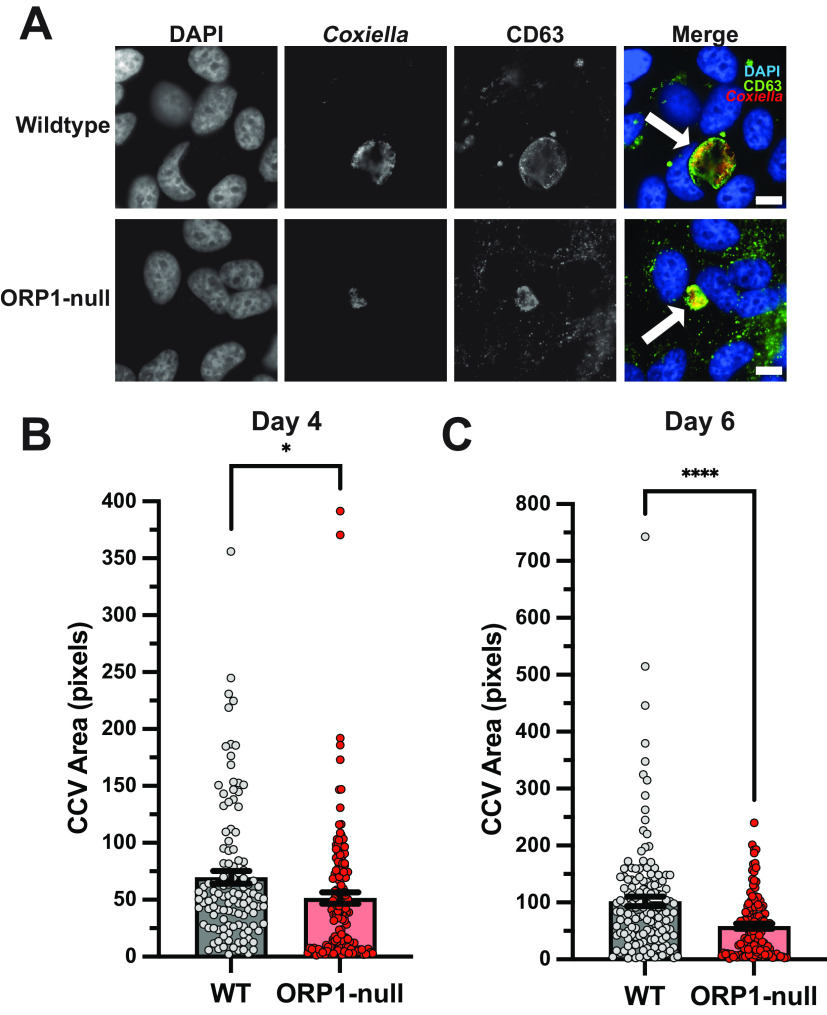
CCV expansion is attenuated in ORP1-null HeLa cells. (A) Representative images of wild-type (WT) and ORP1-null HeLa cells infected with mCherry-expressing C. burnetii for 6 days, then immunofluorescence stained with anti-CD63 antibodies and DAPI. White arrows indicate CCVs. Scale bars, 10 μm. (B) CCV areas in WT and ORP1-null cells after 4 days of infection with mCherry-expressing C. burnetii. WT CCV mean area was 69.65 pixels, and ORP1-null CCV mean area was 51.55 pixels. (C) CCV areas in WT and ORP1-null HeLa cells after 6 days of infection with mCherry-expressing C. burnetii. CCV mean area in WT cells was 101.9 pixels, and ORP1-null CCV mean area was 58.34 pixels. Data shown are means ± standard errors of the mean (SEM) of at least 20 cells per condition in each of three independent experiments as determined by unpaired Student's *t* test. *, *P* < 0.05; ****, *P* < 0.0001.

In a previous study, we showed that the ORP1 isoform ORP1L localizes to the CCV membrane, where it mediates membrane contact sites with the endoplasmic reticulum (13). In order to further elucidate the role of ORP1 during C. burnetii infection, a stable ORP1-null knockout cell line was generated using CRISPR-Cas9 targeting the ORP1 cholesterol-binding ORD in HeLa cells (Fig. 1A). Western blotting confirmed the absence of both ORP1L and ORP1S isoforms (Fig. 1B). Considering ORP1L’s localization to the CCV membrane, we hypothesized that ORP1 is necessary for CCV expansion over a 6-day infection. To determine the effect of ORP1 on CCV development, CCV size was determined in wild-type and ORP1-null HeLa cells at 4 and 6 days postinfection, at which point the CCVs had typically expanded and bacteria were actively replicating (13). Cells were infected with mCherry-expressing C. burnetii and fixed, and the CCV membrane was visualized by immunofluorescence staining for CD63, a host endolysosomal protein that serves as a CCV membrane marker (Fig. 2A). At 4 days postinfection, the mean CCV cross-sectional area in ORP1-null cells was 26.3% smaller than the CCV area in wild-type cells (Fig. 2B). This size difference was more pronounced at 6 days postinfection, where CCVs in ORP1-null cells were 42.8% smaller than CCVs in wild-type cells (Fig. 2C). Notably, wild-type CCVs increased in size by a factor of 1.5 between day 4 and day 6, while ORP1-null CCVs showed little expansion between days 4 and 6 (Fig. 2B and C). These findings suggest that ORP1 promotes CCV expansion during C. burnetii infection.

### ORP1 alters CCV sterol content in HeLa cells.

**FIG 3:**
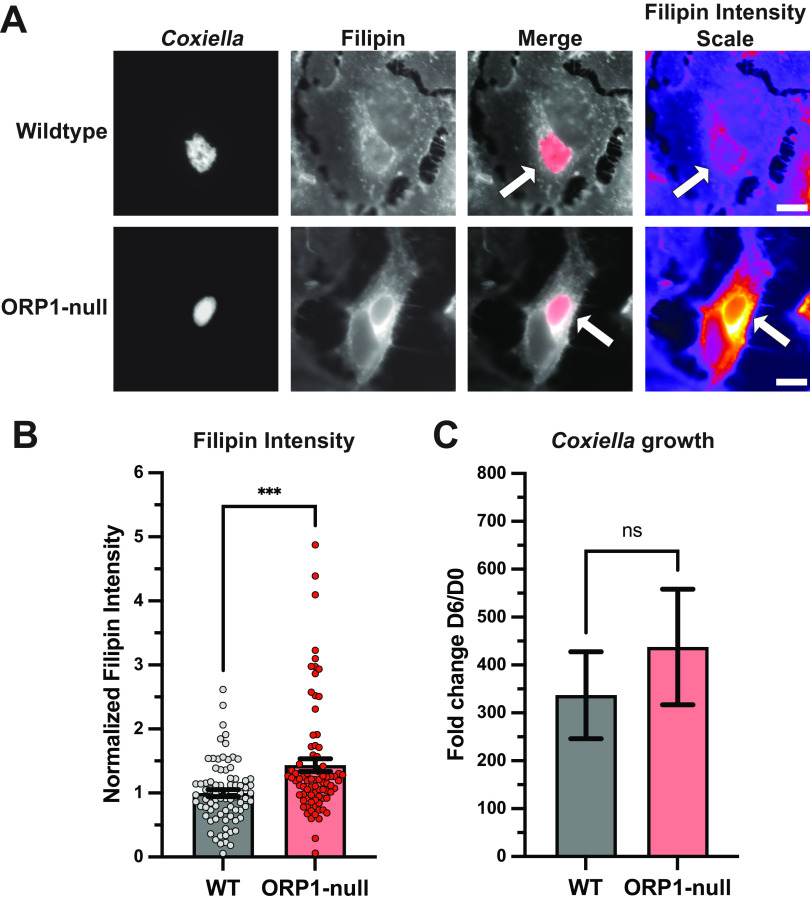
Absence of ORP1 alters CCV sterol content in HeLa cells. (A) Representative images of wild-type and ORP1-null HeLa cells infected with mCherry-expressing C. burnetii for 4 days, then stained with filipin, a fluorescent sterol stain. Filipin intensity scale images show strong filipin signal on the CCV in ORP1-null cells. White arrows indicate CCVs. Scale bars, 10 μm. (B) Filipin intensity in CCVs of HeLa and ORP1-null cells was measured using ImageJ. Images were acquired under identical settings. Intensity values were normalized to area, and background intensity was subtracted from CCV intensity. Data shown are mean ± SEM of at least 20 cells per condition in each of four independent experiments as determined by a nonparametric Mann-Whitney test. ***, *P* < 0.001. (C) C. burnetii growth was determined by CFU assay at 4 days postinfection in wild-type and ORP1-null HeLa cells, then normalized to day zero values to control for initial infection. Data shown are means ± SEM of eight independent experiments; in each independent experiment, each condition was performed at least in duplicate. No statistically significant differences were observed between wild-type HeLa cells and ORP1-null cells at any time point. Data were analyzed using an unpaired two-tailed *t* test.

Considering ORP1’s ability to transfer cholesterol, its promotion of CCV development, and C. burnetii’s need to mitigate cholesterol toxicity, we hypothesized that ORP1 transfers cholesterol away from the CCV. To test this hypothesis, cellular cholesterol was visualized using filipin, a naturally fluorescent compound that binds to unesterified cholesterol (19). Wild-type and ORP1-null HeLa cells were infected with mCherry-expressing C. burnetii, fixed at 4 days postinfection, and then stained with filipin. Fluorescence microscopy was used to quantify filipin fluorescence intensity in the CCV (Fig. 3A and B). CCVs in ORP1-null cells showed 1.4 times higher CCV filipin intensity than CCVs in wild-type cells, suggesting increased sterol content (Fig. 3B). This finding was consistent with the hypothesis that ORP1 promotes CCV expansion by decreasing CCV cholesterol levels.

### ORP1 is not required for C. burnetii growth in HeLa cells.

Due to our observations of limited CCV expansion and increased CCV cholesterol in ORP1-null cells, we hypothesized that C. burnetii would grow poorly in ORP1-null cells compared to wild-type HeLa cells. To determine whether ORP1 is critical for C. burnetii growth, CFU growth assays were done in wild-type and ORP1-null HeLa cells. No significant difference in growth was detected over a 4-day infection (Fig. 3C), indicating that ORP1 is not essential for C. burnetii replication in HeLa cells.

### ORP1 is essential for CCV development and C. burnetii growth in murine alveolar macrophages.

**FIG 4:**
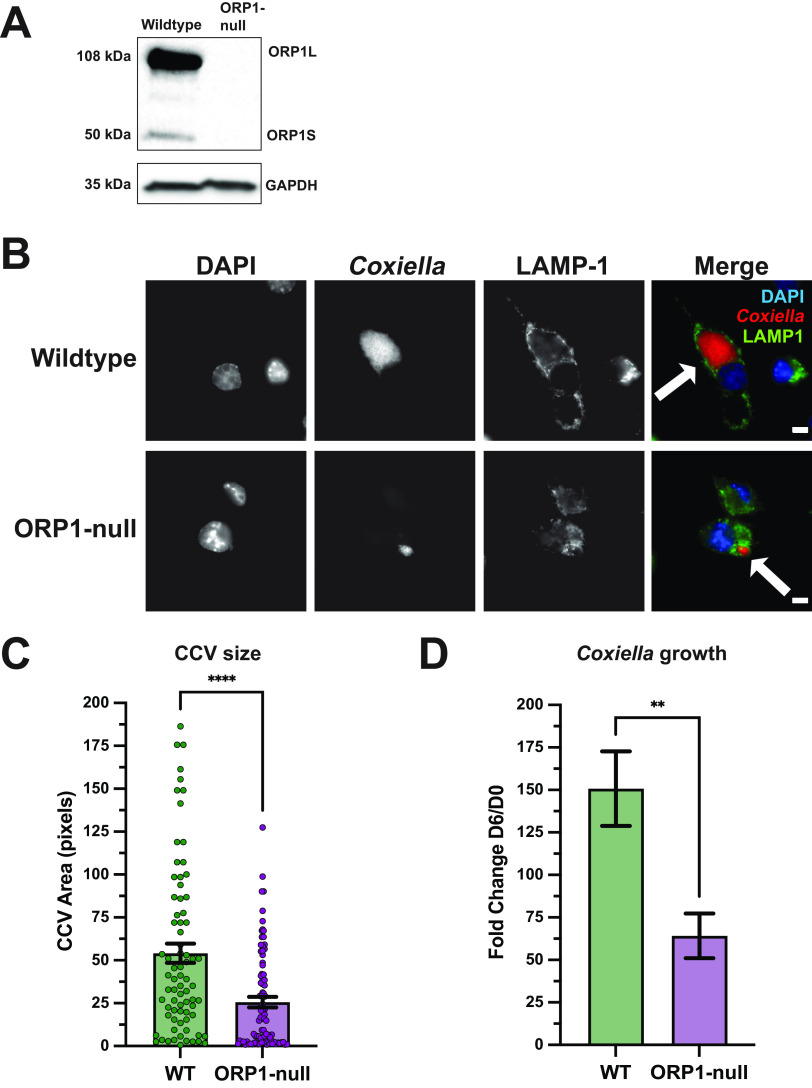
ORP1 is required for full CCV expansion and C. burnetii growth in MH-S macrophages. (A) ORP1 knockout was confirmed by Western blotting. Absence of ORP1L (108 kDa) and ORP1S (50 kDa) was demonstrated in ORP1-null MH-S cells. GAPDH (35 kDa) was used as a loading control. (B) Representative images of wild-type and ORP1-null MH-S cells infected with mCherry-expressing C. burnetii for 6 days, then immunofluorescence stained with anti-LAMP1 antibodies and DAPI. White arrows indicate CCVs. Scale bars, 5 μm. (C) CCV areas in MH-S and ORP1-null cells after 6 days of infection with mCherry-expressing C. burnetii were quantified using ImageJ. Wild-type MH-S mean CCV area was 53.04 pixels, and ORP1-null mean CCV area was 25.62 pixels. Data shown are means ± SEM of at least 19 cells per condition in each of three independent experiments as determined by unpaired Student's *t* test. (D) C. burnetii growth was determined by counting CFU at 6 days postinfection in wild-type and ORP1-null MH-S macrophages, then normalizing values to day zero to control for initial infection. Data shown are means ± SEM of six independent experiments; in each independent experiment, each condition was performed in duplicate. Data were analyzed using an unpaired two-tailed *t* test. **, *P* < 0.01; ****, *P* < 0.0001.

While the absence of ORP1 in HeLa cells affected CCV expansion and CCV cholesterol levels, no difference in growth was detected between ORP1-null and wild-type HeLa cells. During natural infection, C. burnetii targets lung alveolar macrophages. Macrophages are cholesterol rich and increase ORP1 expression upon differentiation from monocytes (14); thus, the ORP1-null phenotype may be more distinct and biologically relevant in macrophages than HeLa cells. To this end, CRISPR-Cas9 was used to generate an ORP1-null cell line in MH-S cells, a murine alveolar macrophage cell line. sgRNA targeting the ORD cholesterol binding domain of ORP1 was used to generate a mutant cell line, and the absence of ORP1 was confirmed by Western blotting (Fig. 4A). Importantly, we did not observe a difference in growth between wild-type and ORP1-null MH-S cells (data not shown). ORP1-null MH-S cells were evaluated for CCV size and growth phenotype and compared to wild-type MH-S cells (Fig. 4). As in the HeLa cell model, there was a significant difference in CCV size at 6 days postinfection, with mean CCV area in ORP1-null MH-S cells only half the size of CCV area in wild-type MH-S cells (Fig. 4B and C). Unlike the HeLa cell model, C. burnetii growth was significantly attenuated in ORP1-null MH-S cells, with a 56.8% decrease in growth over 6 days compared to growth of wild-type MH-S cells (Fig. 4D). Together, these data demonstrated that ORP1 is necessary for CCV expansion and C. burnetii growth in MH-S cells.

### ORP1 reduces CCV cholesterol in murine alveolar macrophages.

**FIG 5:**
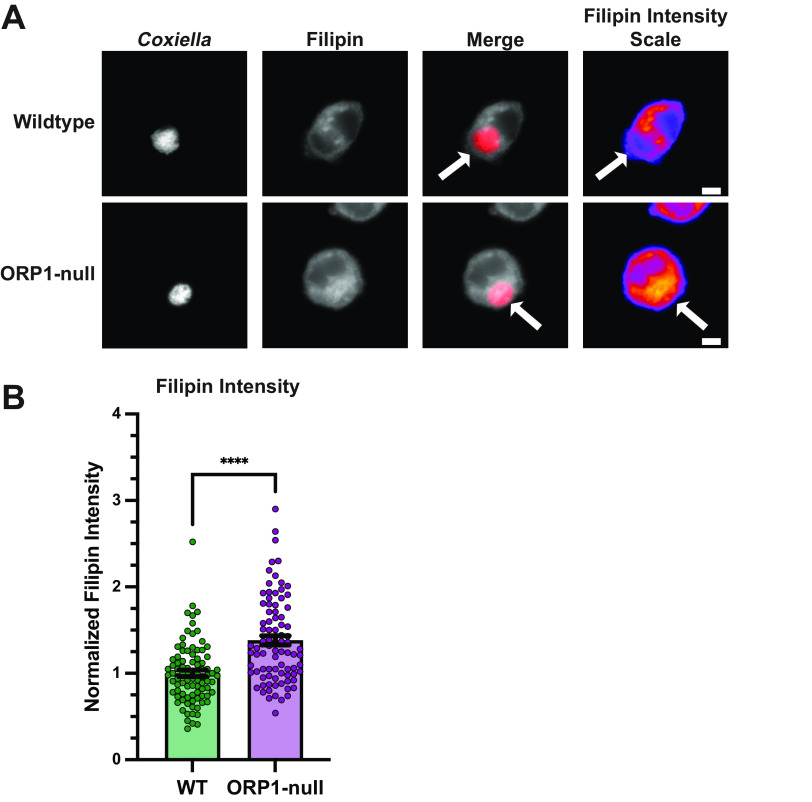
Increased sterol content in the absence of ORP1 in macrophages. (A) Representative images of wild-type and ORP1-null MH-S macrophages infected with mCherry-expressing C. burnetii for 6 days and stained with filipin to visualize cholesterol. Filipin intensity scale images show strong filipin signal on the CCV in ORP1-null macrophages. White arrows indicate CCVs. Scale bars, 10 μm. (B) Filipin intensity in CCVs of WT and ORP1-null macrophages, where intensity values were normalized to area and background intensity was subtracted from CCV intensity. Data shown are means ± SEM of at least 20 cells per condition in each of three independent experiments as determined by a nonparametric Mann-Whitney test. ****, *P* < 0.0001.

Given that ORP1 appears to play a role in decreasing CCV cholesterol in HeLa cells, filipin staining was used to determine cholesterol levels in CCVs from wild-type and ORP1-null alveolar macrophages. As in HeLa cells, we observed an increase in filipin labeling of CCVs in macrophages lacking ORP1 (Fig. 5A and B), suggesting ORP1 is involved in regulating CCV cholesterol in both macrophages and HeLa epithelial cells.

## DISCUSSION

Despite the sterol-rich nature of the CCV, cholesterol is not essential—and in fact inhibits—C. burnetii growth (12). However, C. burnetii readily grows in a variety of cholesterol-rich cells *in vivo* and *in vitro*, suggesting C. burnetii mitigates the toxic effects of cholesterol during host cell infection. We recently demonstrated that C. burnetii recruits the host lipid transport protein ORP1L to the CCV membrane in a T4BSS-dependent manner (13). ORP1L functions in cholesterol transport between LELs and the ER (15, 16), leading to the hypothesis that C. burnetii coopts ORP1 to transport cholesterol away from the CCV, thus forming a CCV more favorable for C. burnetii growth. In this study, we demonstrated that ORP1 is necessary for normal CCV expansion in both HeLa and MH-S cells. Additionally, we found higher CCV sterol content in ORP1-null cells than in wild-type cells, supporting our hypothesis that ORP1 facilitates cholesterol efflux from the CCV. While ORP1 deficiency led to a C. burnetii growth defect in MH-S cells, ORP1 was not required for growth in HeLa cells. This suggested that ORP1’s impact on C. burnetii growth is cell type specific and that compensatory cholesterol transport mechanisms are sufficient to mitigate the effects of ORP1 absence in HeLa cells.

ORP1 exists in two isoforms: the longer isoform ORP1L, which contains the C-terminal cholesterol binding domain and N-terminal interaction domains, and the shorter isoform ORP1S, which consists of only the cholesterol binding domain (14). ORP1L has been studied extensively in the context of cellular cholesterol homeostasis. In mammalian cells, ORP1L primarily localizes to the LEL membrane through interactions between its N-terminal ankyrin repeat domains and Rab7 on LELs (20). ORP1L forms MCSs between LELs and the ER through interaction between its FFAT (two phenylalanines in an acidic tract) motif and the ER VAP proteins (16). Through LEL-ER membrane contact sites, ORP1L promotes cholesterol transfer, and ORP1L-deficient cells have a significant defect in cholesterol efflux from LELs (15, 21). However, it is unclear whether ORP1L directly binds and transfers cholesterol at the MCS or if ORP1L’s function is to maintain the MCS while other proteins transport cholesterol. In the context of C. burnetii infection, ORP1 promotes cholesterol efflux from the CCV, but as with LEL-ER membrane contact sites, ORP1 may serve as a tether between the CCV and ER rather than actively transporting cholesterol. One potential alternative cholesterol efflux mechanism is through ORP5, another member the ORP lipid transport protein family. ORP5 localizes to the ER, where it can interact with NPC1 on LELs, promoting cholesterol transport from LELs to the ER (22). The role of ORP5 and other ORP family members during C. burnetii infection is unknown.

When considering the role of ORP1L in C. burnetii infection, it is important to note that ORP1L not only functions as a cholesterol transporter but also regulates endosome positioning through a cholesterol-dependent mechanism. In high-cholesterol endosomes, ORP1L adopts a conformation that allows recruitment of the dynein-dynactin motor subunit p150^Glued^ to endosomes and promotes minus-end transport (16, 20, 23, 24). Under low-cholesterol conditions, conformation changes allow the ORP1L FFAT motif to bind VAP on the ER, forming endosome-ER membrane contact sites and preventing minus-end transport of endosomes (16, 20, 23, 24). Thus, high-cholesterol conditions cause ORP1L-positive endosomes to cluster near the middle of the cell at the microtubule organizing center, while low-cholesterol conditions cause ORP1L-positive endosomes to remain scattered throughout the cell periphery, tethered to the ER (16, 18, 24). Considering ORP1L’s effect on endosome positioning and the fact that the CCV readily interacts with lysosomes, endosomes, and autophagosomes, it is possible that CCVs in ORP1-null cells fail to expand due to a disruption in endosome motility and CCV-endosome fusion. However, ORP1L’s effect on host endosome positioning would not explain our previous discovery of C. burnetii’s active recruitment of ORP1L to the CCV membrane (13). Additionally, the CCV is not always in the same position inside the host cell; it can be found near the nucleus or on the cell periphery, meaning that ORP1L’s effect on endosome positioning may not affect the CCV’s ability to interact with other vesicles.

The function of ORP1S, the shorter isoform of ORP1, is poorly understood. ORP1S does not localize to endosomes and lysosomes like ORP1L but primarily localizes to the cytoplasm (14). ORP1S can translocate to the nucleus, where it binds to liver X receptor (LXR) transcription factors, enhancing transcription of LXR-dependent genes, such as ApoE (apolipoprotein E) (14, 25). A recent study found evidence that ORP1S functions in cholesterol transport from LELs to the plasma membrane (21). Zhao et al. proposed a model of cellular cholesterol transport in which both ORP1L and ORP1S facilitated cholesterol efflux from LELs, with ORP1L transporting LEL cholesterol to the ER and ORP1S transporting LEL cholesterol to the plasma membrane (21). During C. burnetii infection, the ER seems a more likely recipient of CCV cholesterol, considering our previous finding of increased ER-derived lipid droplets in infected MH-S cells (26). However, we have not yet investigated C. burnetii’s effect on plasma membrane cholesterol, so we cannot rule out the possibility that ORP1S plays a role in CCV cholesterol efflux. The specific roles of ORP1L versus ORP1S in C. burnetii infection are outside the context of this study but present an intriguing avenue for future research.

In conclusion, our results show that ORP1 functions to promote expansion of the CCV during C. burnetii infection. Additionally, we have provided evidence that ORP1 facilitates cholesterol efflux from the CCV and promotes bacterial growth in macrophages. This study is the first to investigate ORP1’s effect on C. burnetii in an alveolar macrophage cell line (MH-S cells). Our findings demonstrate the complex nature of host cell lipid transport manipulation by intracellular pathogens such as C. burnetii. Future investigations will further elucidate the relationship between C. burnetii infection, ORP1, and intracellular cholesterol transport by comparing the specific effects of isoforms ORP1L and ORP1S on C. burnetii.

## MATERIALS AND METHODS

### Bacterial and mammalian cell culture.

Human cervical epithelial cells (HeLa; ATCC CCL-2; ATCC, Manassas, VA, USA) and mouse alveolar macrophages (MH-S; ATCC CRL-2019) were incubated at 37°C in 5% CO_2_ and maintained in RPMI (Roswell Park Memorial Institute) 1640 medium (Corning, New York, NY, USA) with 10% fetal bovine serum (FBS; Atlanta Biologicals, Norcross, GA, USA) and 2 mM l-alanyl-l-glutamine (Glutagro; Corning). Human embryonic kidney cells (HEK 293T; ATCC CRL-3216) were incubated at 37°C in 5% CO_2_ and maintained in Dulbecco’s modified Eagle medium (DMEM; Corning) with 10% FBS. All mammalian cell cultures were passaged every 3 days, and no cells older than 20 passages were used in experiments. Coxiella burnetii Nine Mile phase II (NMII; clone 4, RSA 439) expressing mCherry was grown for 28 days in Vero cells, washed with phosphate-buffered saline (PBS), and stored as previously described (27). For each mammalian cell type, bacterial multiplicity of infection (MOI) was optimized so that no more than one bacterium was internalized in each infected cell.

### Generation of ORP1-null cells using CRISPR-Cas9.

Human ORP1-targeted sgRNA Cas9 plasmids were obtained from GeneCopoeia (HCP291905-SG01-3-B, 3× sgRNA expression clones targeting OSBPL1A; accession number NM_018030.4; Rockville, MD, USA). HeLa cells were transfected with plasmid containing Cas9 and sgRNA sequence CCTGTGTTTCTTGATGCCAT (targeting the region coding for amino acid 528 of ORP1L and amino acid 15 ORP1S) using FuGENE 6 transfection reagent (Promega, Madison, WI, USA). Two days after transfection, cells were placed under 200-μg/mL hygromycin selection. Hygromycin was removed after 4 days, and surviving cells were cloned out by limiting dilution. Clonal populations were expanded and screened for ORP1 expression by Western blotting.

MH-S ORP1-null cells were generated using transduction with lentivirus expressing sgRNA and Cas9. An ORP1-sgRNA was inserted into lentiCRISPRv2 plasmid, which expresses Cas9 (Addgene plasmid number 52961; Watertown, MA, USA). The sgRNA target sequence used was CTGAGATGAGCCTAAACCCA, targeting ORP1 amino acid 640. HEK 293T cells were transfected with the sgRNA-lentiCRISPRv2 plasmids and lentivirus packaging plasmids using FuGENE 6 transfection agent (Promega). Lentivirus-containing supernatant was collected from transfected cells at 24, 48, and 72 h postransduction, combined, and concentrated using Lenti-X concentrator (TaKaRa Bio USA, San Jose, CA, USA), and lentivirus particle concentration was quantified using a Lenti-X reverse transcription-quantitative PCR titration kit (TaKaRa Bio USA). MH-S cells were transduced with lentivirus, and 24 h after transduction, MH-S cells were placed under 5-μg/mL puromycin selection for 10 days, and then surviving cells were cloned out by limiting dilution. Clonal populations were expanded and screened for ORP1 expression by Western blotting.

### SDS-PAGE and Western blotting.

Potential mutant clones and parental control cells were lysed with 2% sodium dodecyl sulfate (SDS). Protein concentration of samples was measured using Pierce bicinchoninic acid assay kit (Thermo Fisher Scientific, Waltham, MA, USA), and 20 μg of each sample was loaded on a 4-to-20% SDS-PAGE gel (Bio-Rad Laboratories, Hercules, CA, USA). Protein was transferred to a nitrocellulose membrane using the Trans-Blot Turbo transfer system (Bio-Rad Laboratories), and the membrane was blocked in 5% milk in Tris-buffered saline with 0.1% Tween (TBS-T; Sigma-Aldrich, St. Louis, MO, USA) for 1 h. After blocking, the membrane was probed with primary antibody for either ORP1 (1:1,000; Abcam ab131165; Cambridge, United Kingdom) or glyceraldehyde 3-phosphate dehydrogenase (GAPDH) loading control (1:2,500; Invitrogen MA5-15738; Waltham, MA, USA) in a solution of 1% bovine serum albumin (BSA) in PBS overnight. The ORP1 antibody ab131165 target sequence is proprietary, but this antibody detects both ORP1L and ORP1S variants. The membrane was washed in TBS-T, then incubated in secondary antibody mixture (horseradish peroxidase [HRP]-Strep anti-rabbit and HRP-Strep anti-mouse, both diluted 1:2,500 in 5% milk). After secondary antibody incubation, membranes were washed in TBS-T, then treated with SuperSignal chemiluminescent substrate (Thermo Fisher Scientific) and imaged using an Azure 280 gel imager (Azure Biosystems, Dublin, CA, USA).

### Quantification of CCV area.

HeLa cells were plated at 2 × 10^5^ cells per well in a 6-well tissue culture plate and allowed to adhere overnight. Cells were infected with mCherry-expressing C. burnetii in 500 μL 10% RPMI for 2 h, then washed 15 times with PBS to remove extracellular bacteria. At 3 and 5 days postinfection, cells were plated on coverslips in a 24-well plate, in triplicate, at 5 × 10^4^ per well and allowed to adhere overnight. The following day (time points 4 and 6 days postinfection), coverslips were fixed with 2.5% formaldehyde in PBS for 15 min, permeabilized with 0.1% saponin in 1% BSA in PBS for 20 min, and then washed in PBS. Coverslips were incubated for 1 h with mouse anti-CD63 primary antibody (1:1,000; catalog number 556019, BD Biosciences, Franklin Lakes, NJ, USA) in 1% BSA in PBS. Coverslips were washed in PBS, then incubated for 30 min with goat anti-rabbit IgG cross-adsorbed secondary antibody Alexa Fluor 488 (Invitrogen) diluted 1:1,000 in 1% BSA in PBS. Coverslips were washed in PBS, then mounted to microscope slides using ProLong Gold antifade mountant with 4′,6-diamidino-2-phenylindole (DAPI; Thermo Fisher Scientific) and allowed to dry overnight. Coverslips were imaged on a Nikon Eclipse Ti-E microscope using a 63× oil objective (1.4 numerical aperture [NA]). Images were processed using Fiji Image J software, with at least 20 CCVs measured for each condition across three independent experiments (28). Data were analyzed by Student's *t* test using GraphPad Prism 9 software. For MH-S cells, the above protocol was followed, except instead of staining with anti-CD63 antibody, cells were stained with rabbit anti-LAMP1 antibody (Abcam ab24170), and images were taken using a Leica epifluorescence microscope using a 100× oil objective (1.4 NA).

### Filipin labeling and imaging.

HeLa cells were plated at 2 × 10^5^ cells per well in a 6-well tissue culture plate and allowed to adhere overnight. MH-S macrophages were plated at 3 × 10^5^ cells per well in a 6-well tissue culture plate and allowed to adhere overnight. Cells were infected with mCherry-expressing C. burnetii in 500 μL 10% RPMI for 2 h, then washed with PBS to remove extracellular bacteria. At 3 days postinfection, HeLa cells were replated onto coverslips in a 24-well plate at 5 × 10^4^ cells/well and allowed to adhere overnight. At 4 days postinfection, MH-S cells were plated onto coverslips at 5 × 10^4^ and allowed to adhere for 1 day. The following day, cells were fixed in 2.5% formaldehyde in PBS on ice for 15 min, then incubated for 1 h in the dark with filipin (Cayman Chemicals, Ann Arbor, MI, USA; 5 mg/mL stock in dimethyl sulfoxide) diluted 1:100 in 1% BSA in PBS. After incubation, coverslips were washed with PBS and mounted onto microscope slides with ProLong Gold mounting medium. Images were acquired using a Zeiss epifluorescence microscope using a 60× oil objective. For MH-S filipin imaging, images were acquired using a Nikon Ti2 epifluorescence microscope and 100× oil objective. Within each experiment, images were captured under identical settings and processed using Fiji ImageJ. In Fiji ImageJ, filipin fluorescence in the CCV was determined by quantitating mean gray value within the CCV, then subtracting the background mean gray value. At least 20 CCVs were measured per condition across four independent experiments. Data were normalized to average fluorescence intensity of the control HeLa condition in order to account for variability between independent experiments, and conditions were compared using the Kruskal-Wallis nonparametric test.

### Quantification of C. burnetii growth by CFU assay.

CFU assays were performed as previously described (29). HeLa cells were plated in duplicate or triplicate in a 6-well plate at 2 × 10^5^ per well and allowed to adhere overnight. The next day, cells were infected with mCherry-expressing C. burnetii in 500 μL 10% RPMI for 2 h, then washed 15 times with PBS to remove extracellular bacteria. Cells were scraped into 2 mL fresh 10% RPMI. Infected cells from each condition were replated in a 24-well plate for analysis at day 2 (500 μL cells), day 4 (250 μL cells + 250 μL 10% RPMI), and day 6 (125 μL cells + 325 μL 10% RPMI) postinfection. To establish a baseline number of internalized bacteria at day zero, 500 μL of infected cells was centrifuged into a pellet and then lysed in sterile water for 5 min. Lysate was serially diluted in ACCM-D and spotted onto ACCM-D agarose plates, with each condition performed in triplicate. For all other time points, plated cells were lysed in the 24-well plate in sterile water for 5 min. ACCM-D agarose plates were incubated for 7 to 10 days at 37°C in 2.5% O_2_ and 5% CO_2_, and the number of colonies was counted to determine bacterial viability. For each time point, fold change over day zero was determined. Eight independent experiments were performed, each in biological duplicates or triplicates. For CFU assays in MH-S cells, the same protocol was followed with the following exception: at days 2, 4, and 6, cells were lifted from 24-well plates with trypsin. Trypsin was deactivated with 10% RPMI, and the cells were centrifuged and lysed as for the day zero HeLa cells described above.

### Statistical analyses.

All statistical analyses were performed using GraphPad Prism 9 software.

### Data availability.

Data associated with this project will be uploaded into Zenodo (https://doi.org/10.5281/zenodo.7677894).
